# Respiratory Support in Meconium Aspiration Syndrome: A Practical Guide

**DOI:** 10.1155/2012/965159

**Published:** 2012-02-23

**Authors:** Peter A. Dargaville

**Affiliations:** ^1^Department of Paediatrics, Royal Hobart Hospital and University of Tasmania, Hobart, TAS 7000, Australia; ^2^Neonatal Respiratory Group, Menzies Research Institute, Hobart, TAS 7000, Australia

## Abstract

Meconium aspiration syndrome (MAS) is a complex respiratory disease of the term and near-term neonate. Inhalation of meconium causes airway obstruction, atelectasis, epithelial injury, surfactant inhibition, and pulmonary hypertension, the chief clinical manifestations of which are hypoxaemia and poor lung compliance. Supplemental oxygen is the mainstay of therapy for MAS, with around one-third of infants requiring intubation and mechanical ventilation. For those ventilated, high ventilator pressures, as well as a relatively long inspiratory time and slow ventilator rate, may be necessary to achieve adequate oxygenation. High-frequency ventilation may offer a benefit in infants with refractory hypoxaemia and/or gas trapping. Inhaled nitric oxide is effective in those with pulmonary hypertension, and other adjunctive therapies, including surfactant administration and lung lavage, should be considered in selected cases. With judicious use of available modes of ventilation and adjunctive therapies, infants with even the most severe MAS can usually be supported through the disease, with an acceptably low risk of short- and long-term morbidities.

## 1. Introduction

Meconium aspiration syndrome (MAS) is complex respiratory disease of the term and near-term neonate that continues to place a considerable burden on neonatal intensive care resources worldwide. The condition has features that make it stand alone amongst neonatal respiratory diseases—the unique combination of airflow obstruction, atelectasis, and lung inflammation, the high risk of coexistent pulmonary hypertension, and the fact of these occurring in a term infant with a relatively mature lung structurally and biochemically. For all these reasons, management of MAS, and in particular the ventilatory management of MAS, has been a difficult challenge for neonatologists down the years. This paper focuses on application of mechanical respiratory support in MAS, as well as the role of adjunctive respiratory therapies. For the purpose of the paper, MAS is defined as respiratory distress occurring soon after delivery in a meconium-stained infant, which is not otherwise explicable and is associated with a typical radiographic appearance [1]. 

## 2. Pathophysiology and Effects on Gas Exchange and Lung Compliance

Lung dysfunction in MAS is a variable interplay of several pathophysiological disturbances, chief amongst which are airway obstruction, atelectasis, and pulmonary hypertension. Meconium, the viscid pigmented secretion of the fetal intestinal tract [2], is a noxious substance when inhaled, producing one of the worst forms of aspiration pneumonitis encountered in humans. Meconium has many adverse biophysical properties, including high tenacity (stickiness) [3], very high surface tension (215 mN/m) [3], and potent inhibition of surfactant function [4–6]. It is also directly toxic to the pulmonary epithelium [7], causing a haemorrhagic alveolitis with high concentrations of protein and albumin in the alveolar space [8]. Meconium contains substances that are chemotactic to neutrophils [9] and activate complement [10] and may in addition be vasoactive [11]. These adverse properties of meconium are reflected in the pathophysiological disturbances known to occur in MAS [12].

**Figure 1:**
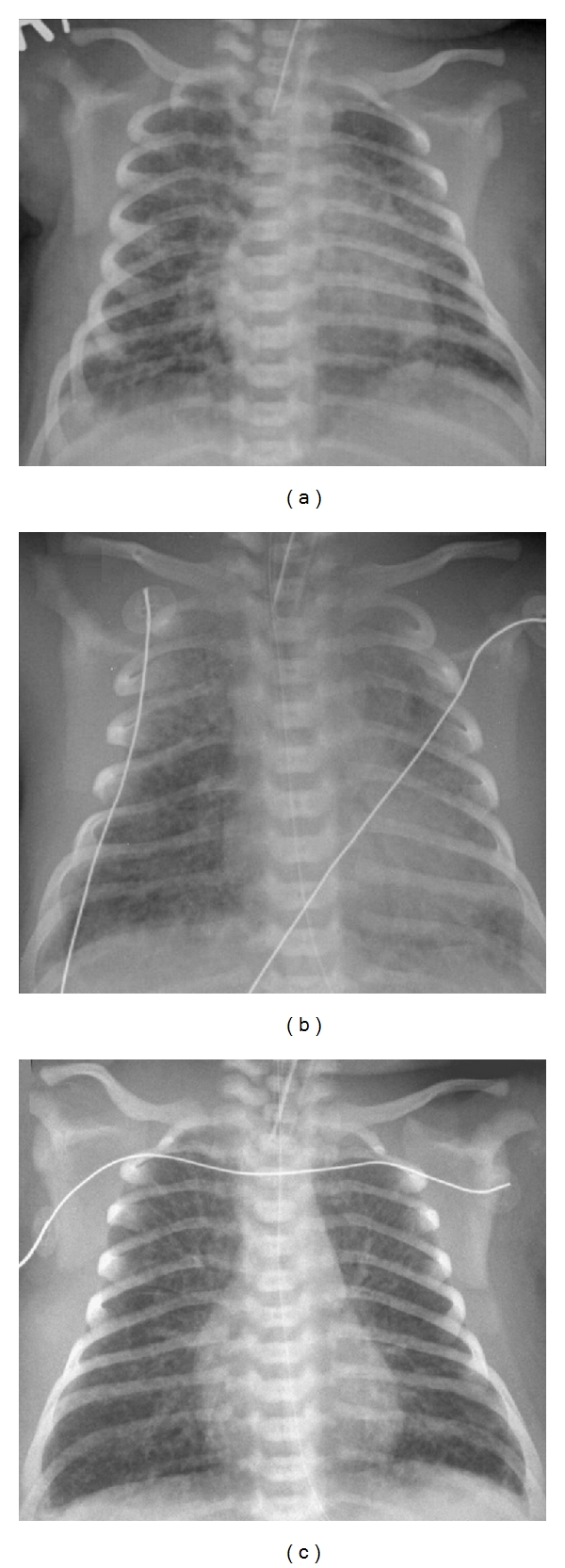
Chest X-ray appearances in ventilated infants with MAS. (a) Typical appearance of MAS showing “fluffy” opacification widespread throughout the lung fields. (b) Marked atelectasis in an infant with profound hypoxaemia. (c) Hyperinflation and gas trapping, with a narrow cardiac waist, flattened diaphragms, and intercostal bulging of the lung.

Once inhaled, migration of meconium down the tracheobronchial tree initially causes obstruction of airways of progressively smaller diameter [13–15]. At least in experimental MAS, there can be a considerable component of “ball-valve” obstruction, with high resistance to airflow in expiration, resulting in gas trapping distal to the obstruction [14]. If global in distribution, high functional residual capacity (FRC) may result, although only in a small proportion of infants with MAS is there measurably high FRC [16, 17], and even then only transiently [17]. For most infants with MAS, the predominant consequence of airway obstruction with meconium is downstream atelectasis [18]. The patchy nature of the airway obstruction results in a juxtaposition of atelectatic and normally aerated lung units, which has been clearly shown histologically [18], and is reflected in the patchy opacification typically noted on chest X-ray in MAS (Figure 1) [19].

After migration to the level of the alveoli, meconium induces a combination of haemorrhagic alveolitis and surfactant inhibition. Meconium is toxic to the alveolar epithelium [7, 20], causing disruption of the alveolocapillary barrier and an exudative oedema not unlike that seen in acute respiratory distress syndrome. The underlying lung interstitium shows inflammatory cell infiltrate [13, 15], and there is a cytokine release in part related to complement activation [10, 21, 22]. Moreover, meconium causes a potent dose-dependent inhibition of surfactant function [4–6] and, along with fibrinogen and haemoglobin in the exudate [23, 24], impairs the capacity of endogenous surfactant to reduce surface tension. Stability of alveoli at end-expiration is thus compromised [25], as is the capacity to clear oedema fluid from the airspaces [26]. The resultant microatelectasis causes variable degrees of ventilation-perfusion mismatch or, worse still, intrapulmonary shunt.

The most prominent and consistent physiological effects resulting from meconium injury are hypoxaemia and decreased lung compliance. Some degree of hypoxaemia is universal in symptomatic MAS, contributed to by many of the above-mentioned noxious effects of meconium. Disturbances of oxygenation in MAS may relate to atelectasis, overdistension, pulmonary hypertension, or a combination of these. A challenging aspect of the management of MAS is to discern which mechanism of hypoxaemia is the predominant one in any given infant at any given time. Particularly where there is prominent airway obstruction or pronounced atelectasis, hypoxaemia may be accompanied by respiratory acidosis with CO_2_ retention related to hypoventilation.

Lung or respiratory system compliance is usually significantly impaired in infants requiring ventilation with MAS [17, 22, 27–30]. Experimental studies have indicated that decreased compliance may be related to hyperinflation secondary to “ball-valve” airway obstruction [14], and the combination of poor compliance and high FRC has been demonstrated in some cases of MAS [17]. For most infants with MAS, in whom FRC is normal or low [17], poor compliance relates to global or regional atelectasis. Application of mechanical ventilation further complicates the picture, potentially leading to overdistension of relatively unaffected lung regions which, due to their relatively long time constant, may empty incompletely during the ventilator expiratory cycle, especially at fast ventilator rates [31]. Respiratory resistance has also been noted to be increased in some studies, but variations in the technique of measurement make interpretation of these results difficult.

MAS is frequently accompanied by persistent pulmonary hypertension of the newborn (PPHN) [32], with many factors contributing to its development, including low pO_2_ and pH, coexistent intrauterine asphyxia, and possibly vasoactive substances in the meconium itself [33].

## 3. Stepwise Approach to Respiratory Support

### 3.1. Oxygen Therapy

Supplemental oxygen administration is the mainstay of treatment for MAS and in many less severe cases is the only therapy required [34]. Some ventilated infants with MAS receive high inspired oxygen concentration for long periods, with few apparent adverse effects. Therapeutic considerations in cases of persistently high oxygen requirement are outlined in Table 1.

As with the preterm infant, moment-by-moment adjustment of oxygen concentration (or flow) in infants with MAS is guided oxygen saturation measured by pulse oximetry (SpO_2_). Given the high incidence of right-to-left ductal shunting related to pulmonary hypertension, a pre-ductal SpO_2_ is preferable, with the target range for SpO_2_ being higher than that for the preterm infant, usually between 94 and 98%. In ventilated infants, oxygen therapy can also be monitored by blood gas sampling from an intra-arterial line, preferably in a preductal position in the right radial artery. Suggested target pO_2_ range is 60–100 mm Hg (preductal). Where there is considerable PPHN, titration of FiO_2_ using postductal pO_2_ values is not advisable.

Approach to hypoxaemia in MAS.

### 3.2. Continuous Positive Airway Pressure

Of all infants requiring mechanical respiratory support because of MAS, approximately 10–20% are treated with continuous positive airway pressure (CPAP) alone [34–36]. Additionally, up to one-quarter of infants requiring intubation with MAS receive CPAP before and/or after their period of ventilation [36]. CPAP for such infants can be effectively delivered by binasal prongs or a single nasal prong, typically with a CPAP pressure of 5–8 cm H_2_O. Tolerance of the CPAP device may be limited given the relative maturity of infants with MAS, and on occasions the associated discomfort will exacerbate pulmonary hypertension to the point where intubation becomes necessary.

### 3.3. Intubation

Approximately one-third of all infants with a diagnosis of MAS require intubation and mechanical ventilation [33, 37]. Indications for intubation of infants with MAS include (a) high oxygen requirement (FiO_2_ > 0.8), (b) respiratory acidosis, with arterial pH persistently less than 7.25, (c) pulmonary hypertension, and (d) circulatory compromise, with poor systemic blood pressure and perfusion [38]. Except in emergency circumstances, intubation of infants with MAS should be performed with premedication. Significant endotracheal tube leak is a major barrier to effective ventilation in infants with MAS, and in most cases a size 3.5 mm internal diameter endotracheal tube will be required. Once intubated, tolerance of the endotracheal tube will almost certainly require ongoing sedation with infusions of an opiate (e.g., morphine or fentanyl) [39], possibly supplemented with a benzodiazepine. Additionally, continuation of muscle relaxant drugs is often helpful during the stabilisation period after intubation, particularly in infants with coexistent pulmonary hypertension.

### 3.4. Conventional Mechanical Ventilation

Despite more than four decades of mechanical ventilation for infants with MAS, the ventilatory management of the condition remains largely in the realm of “art” rather than “science”, with very few clinical trials upon which to base definitive recommendations. Physiological principles and published experience do, however, allow some guiding principles to be put forward for conventional ventilation strategy in MAS.

#### 3.4.1. Choosing a Mode of Ventilation

Ventilation mode and the value of patient-triggering have been incompletely studied in MAS. Two randomised trials of patient-triggered ventilation have included infants with MAS. One of these found no advantage of synchronised intermittent mandatory ventilation (SIMV) over IMV in 15 infants with MAS [40]. Another study found, in a group of 93 infants >2 kg birth weight (including an unspecified number with MAS), that use of SIMV was associated with a shorter duration of ventilation compared with IMV [41]. Despite the relative paucity of evidence in favour, it seems logical to use a synchronized mode of ventilation in any spontaneously breathing ventilated infant with MAS. Trigger sensitivity should be set somewhat higher than that for a preterm infant and should take into account the possibility of autocycling if there is a tube leak [42]. There have been no clinical trials in patients with MAS comparing SIMV and synchronised intermittent positive pressure ventilation (SIPPV), also known as assist control. Given the propensity for gas trapping in MAS, there is some concern that using SIPPV may lead to high levels of inadvertent positive end-expiratory pressure (PEEP) with resultant hyperinflation. For this reason SIMV may be the most appropriate mode of ventilation in MAS.

#### 3.4.2. Selection of Positive End-Expiratory Pressure

For any newborn respiratory disease, but particularly MAS, application of PEEP must balance the competing interests of overcoming atelectasis whilst avoiding overdistension. Early observations of the effect of PEEP suggested the greatest benefit with PEEP settings between 4 and 7 cm H_2_O, with higher PEEP settings (8–14 cm H_2_O) giving minimal oxygenation benefit [43]. No more recent clinical studies exist to guide PEEP selection in MAS. Physiological principles dictate that if atelectasis predominates (Figure 1(b)), increasing PEEP (up to a maximum of 10 cm H_2_O) should improve oxygenation, whereas for regional or global hyperinflation (Figure 1(c)) a lower PEEP (3-4 cm H_2_O) may be effective (Table 1) [38]. For infants with severe atelectasis, PEEP settings above 10 cm H_2_O are likely to increase the risk of pneumothorax [44], and modes of high frequency ventilation are to be preferred if available.

#### 3.4.3. Selection of Inspiratory Time

As with PEEP, setting inspiratory time in MAS must take into account the balance between atelectasis and overdistension. Term infants have generally longer time constants than their preterm counterparts [45] and thus require a longer inspiratory time (around 0.5 sec) to allow near-full equilibration of lung volume change in response to the applied peak pressure. Even longer inspiratory times may be useful for lung recruitment during inspiration if atelectasis is prominent.

#### 3.4.4. Selection of Peak Inspiratory Pressure (or Tidal Volume)

Given the reduced compliance, the peak inspiratory pressure (PIP) required to generate sufficient tidal volume in MAS is often high (30 cm H_2_O or more). Such pressures may well contribute to a secondary ventilator-induced lung injury in ventilated infants with MAS. Suggested target tidal volume is 5-6 mL/kg. If using a “volume guarantee” mode, the peak pressure limit should be set at or near 30 cm H_2_O to allow the ventilator to scale up the PIP when needed to reach the tidal volume target. If PIP is persistently higher than 30 cm H_2_O, high frequency ventilation should be considered, if available.

#### 3.4.5. Selection of Ventilator Rate

Especially if there is gas-trapping and expiratory airflow limitation, optimal conventional ventilation in MAS requires the use of a relatively low ventilator rate (<50) and hence longer expiratory time. This will help to avoid inadvertent PEEP. The resultant minute ventilation must be sufficient to produce adequate CO_2_ clearance. An acceptable arterial pCO_2_ range is 40–60 mm Hg and pH 7.3-7.4, which is achievable in most infants even when there is significant parenchymal disease combined with PPHN [46]. Hyperventilation-induced alkalosis, which anecdotally appeared to reduce the need for extracorporeal membrane oxygenation (ECMO) in infants with PPHN [47], is no longer practiced, in part due to the risk of sensorineural hearing loss [48].

### 3.5. High-Frequency Oscillatory Ventilation

Despite the dearth of clinical trial, evidence suggesting a benefit, high frequency oscillatory ventilation (HFOV) has become an important means of providing respiratory support for infants with severe MAS failing conventional ventilation. Published series from large neonatal databases suggest that 20–30% of all infants requiring intubation and ventilation with MAS are treated with high-frequency ventilation [34, 36, 49], with most of these receiving HFOV rather than high-frequency jet ventilation (HFJV). Indications for transitioning to HFOV include ongoing hypoxaemia and/or high FiO_2_, and, less commonly, respiratory acidosis. In infants with significant atelectasis, adequate lung recruitment may require the application of a mean airway pressure (*P*_AW_) considerably higher than that on conventional ventilation (up to 25 cm H_2_O in some cases), with a stepwise recruitment manoeuvre likely to be the most effective [50]. Once oxygenation has improved, *P*_AW_ should then be reduced; most infants with MAS requiring HFOV can be stabilised using a *P*_AW_ around 16–20 cm H_2_O, with gradual weaning in the days thereafter [51]. Infants with prominent gas trapping may tolerate the recruitment process poorly, with reductions in oxygenation and systemic blood pressure and the potential for exacerbation of pulmonary hypertension. Recruitment manoeuvres of some form can still be advantageous in this group, with the benefit becoming apparent when the *P*_AW_ is reduced.

Choice of oscillatory frequency is critically important in MAS, with experimental studies and clinical experience indicating that frequency should not be higher than 10 Hz and preferably should be set at 8 or even 6 Hz. In experimental models of MAS, high oscillatory frequency (15 Hz) is associated with worsening of gas trapping [52]. HFOV can also lend a clinical advantage in infants with significant coexisting PPHN, as the response to inhaled nitric oxide (iNO) is better when delivered on HFOV compared to conventional ventilation [53]. Early reports suggested that up to half of infants with MAS treated with HFOV did not respond adequately and went on to receive ECMO [54, 55]. More recent experience would suggest that only around 5% of infants treated with HFOV and iNO fail to respond and undergo transition to ECMO [36]. 

### 3.6. High-Frequency Jet Ventilation

The combination of atelectasis and gas trapping that can occur in MAS may be better managed with HFJV than HFOV (Table 1), with the former technique offering the possibility of ventilation at a lower *P*_AW_ [56]. A number of laboratory investigations have shown HFJV, either alone or in combination with surfactant therapy, to be beneficial in animal models of MAS [18, 56, 57]. Clinical studies including infants with MAS appear to confirm the benefit of HFJV compared with conventional ventilation, both in terms of improvement in oxygenation, and avoidance of ECMO [58, 59]. Whilst there have been no direct comparisons with HFOV in a clinical setting, we have noted that some infants with intractable hypoxaemia and/or respiratory acidosis do show improvements after transition from HFOV to HFJV using a low-frequency (240–360 bpm) and a low conventional ventilator rate [60].

## 4. Adjunctive Respiratory Therapies

### 4.1. Bolus Surfactant Therapy

The pathophysiology of MAS includes inhibition of surfactant in the airspaces, both by meconium and exuded plasma proteins [4–6, 23]. Preliminary reports of the use of exogenous surfactant given as bolus therapy to ventilated infants with MAS were promising, although it was identified that around 40% of cases did not respond [61]. Four randomised controlled trials of bolus surfactant therapy have been conducted [62–65], which when analysed together show a benefit in terms of reduction in need for ECMO but not duration of ventilation or other pulmonary outcomes [66]. In the developed world, bolus surfactant therapy is currently used in 30–50% of ventilated infants with MAS [34, 36]. Bolus surfactant therapy should be used judiciously in MAS, choosing infants with severe disease, and treating early and, if necessary, repeatedly [12].

### 4.2. Lavage Therapy

Lung lavage using dilute surfactant is an emerging treatment for MAS that offers the potential of interrupting the pathogenesis of the disease by removal of meconium from the airspaces [12]. Laboratory studies and preliminary clinical evaluations have indicated that lavage therapy may improve oxygenation and shorten duration of ventilation in MAS [67–69]. A recent randomised controlled trial of large-volume lavage using dilute surfactant in infants with severe MAS noted no effect on duration of respiratory support or other pulmonary outcomes but did find a higher rate of ECMO-free survival in the treated group [70]. Further clinical trials will be necessary to more precisely define the effect on survival.

### 4.3. Corticosteroid Therapy

Steroid therapy has been investigated in MAS for more than 3 decades, with a number of small clinical trials being conducted, none of which have given a definitive result. One recent trial suggested that dexamethasone therapy could dampen the inflammatory response in MAS [71]. In the absence of further trials, steroid therapy cannot be recommended as routine therapy in MAS.

### 4.4. Inhaled Nitric Oxide

Large randomised controlled trials have demonstrated the effectiveness of iNO in term infants with pulmonary hypertension, with a reduction in need for ECMO and in the composite outcome of death or requirement for ECMO [72]. Each trial included a large subgroup with MAS; overall more than 640 infants with MAS have been enrolled in iNO trials, although few have reported the outcome for MAS separately. The potential value of delivering iNO during HFOV has been highlighted in one trial, in which the proportion of nonresponders was lowest when the two therapies were combined [53]. Currently around 20–30% of all ventilated infants with MAS receive iNO [34, 36], and around 40–60% show a sustained response [46, 53].

The approach to an infant with MAS and coexistent PPHN should initially focus on optimising the ventilatory management and in particular overcoming atelectasis whilst avoiding hyperinflation, both of which are associated with an increase in pulmonary vascular resistance. The severity of PPHN should be assessed clinically and by echocardiogram if available. If moderate-severe PPHN persists after appropriate ventilatory manoeuvres and the pO_2_ remains at less than 80–100 mm Hg in FiO_2_ 1.0 [53, 73], iNO should commence at a dose of 10–20 ppm. Higher doses do not appear to result in better oxygenation [74].

### 4.5. Extracorporeal Membrane Oxygenation

Infants with severe MAS have been treated with ECMO since 1976, and MAS has been the leading diagnosis amongst neonates referred for this therapy [75]. ECMO is now available to infants with MAS in selected centres in 33 countries worldwide [76], albeit at a high cost (at least 2.5 times the daily cost of standard intensive care) [77]. With the advent of newer therapies, the number of infants with MAS treated with ECMO has decreased [78], but survival with ECMO treatment for MAS has remained high (around 95%) [75]. The usual indication for commencing ECMO is intractable hypoxaemia despite optimisation of the patient's condition with available therapies (including high-frequency ventilation and iNO) and bolus surfactant therapy). Degree of hypoxaemia in this setting has generally been quantified using oxygenation index (OI), where OI = *P*_AW_ × FiO_2_ × 100/PaO_2_. An OI persistently above 40 despite aggressive standard management has been, and remains, an indication for treatment with ECMO where available [79]. Followup of newborn infants treated with ECMO because of parenchymal lung disease (excluding diaphragmatic hernia) suggests a low rate of severe disability at one year (1.7% in the UK ECMO trial) [80], with the risk of any disability being 17% [80].

### 4.6. Liquid Ventilation

To the author's knowledge, there is as yet no report of clinical use of perfluorocarbon in MAS. Both total liquid ventilation with perfluorocarbon and perfluorocarbon-assisted gas exchange have been investigated in animal models of MAS [81–83]. Both techniques have shown short-term advantages over conventional ventilatory management, with better oxygenation and lung compliance [81, 83]. Total liquid ventilation appears to be the most lung protective, resulting in much reduced meconium-associated histological damage compared with conventional ventilation or PAGE [81]. The complications of perfluorocarbon instillation noted in human subjects, including pneumothorax, impaired carbon dioxide clearance, and delayed excretion, may be significant barriers to the clinical use of liquid ventilation in ventilated infants with MAS.

Perfluorocarbon has also been considered as a possible vehicle for lung lavage in MAS, especially given the favourable biophysical properties including high oxygen carrying capacity and low surface tension. Despite these potential advantages, use of neither pure [84] nor emulsified [69] perfluorocarbon as a lavage fluid has shown any major advantage over dilute surfactant. Even when followed by perfluorocarbon-assisted gas exchange, the benefits of perfluorocarbon lavage appear minimal [83]. This may be due to the relatively high density of perfluorocarbon and/or the relative immiscibility of meconium with perfluorochemicals.

## 5. Outcome of Ventilation in MAS

### 5.1. Duration of Ventilation and Oxygen Therapy

Considering all intubated infants with MAS, median duration of ventilation is 3 days (mean 4.8 days) [36]. Infants with more severe disease, requiring at least one of high-frequency ventilation, iNO or bolus surfactant, are ventilated for a median of 5 days [36]. Median duration of oxygen therapy and length of hospital stay currently stand at 7 and 17 days, respectively [36].

### 5.2. Mortality

Refinements in intensive care and respiratory support have contributed to a significant decrease in mortality related to MAS, with population-based studies now suggesting a mortality of 1-2 per 100,000 live births [36, 85, 86]. The case-fatality rate in ventilated infants with MAS varies widely in published series (0–37%) [37] and is influenced by availability of alternative means of ventilation, adjunctive therapies including nitric oxide, and ECMO. Approximately one-quarter to one-third of all deaths in ventilated infants with a diagnosis of MAS are directly attributable to the pulmonary disease, with the remainder in large part caused by hypoxic-ischaemic encephalopathy [34, 36, 86].

### 5.3. Short-Term Morbidities

Pneumothorax occurs in around 10% of all ventilated infants with MAS [36, 87], and the presence of this complication potentiates lung atelectasis and PPHN and increases the risk of mortality [36, 88]. Other air leak syndromes, including pneumomediastinum and pulmonary interstitial emphysema, are seen occasionally. Pulmonary haemorrhage (or, more correctly, haemorrhagic pulmonary oedema) occurs in a small proportion of infants with MAS and can occasionally cause severe destabilisation and hypoxaemia [89].

### 5.4. Long-Term Morbidities

Respiratory compromise after hospital discharge is common in infants who were ventilated with MAS. Up to half of infants will be symptomatic with wheezing and coughing in the first year of life [90]. Older children may exhibit evidence of airway obstruction, hyperinflation, and airway hyperreactivity, but appear to have normal aerobic capacity [91]. Neurological sequelae following MAS are well recognized [37], and a diagnosis of MAS in the neonatal period confers a considerable risk of cerebral palsy (5–10%) [92, 93] and global developmental delay (15%) [92].

## 6. Conclusion

With judicious use of available modes of ventilation and adjunctive therapies, infants with even the most severe MAS can usually be supported through the disease, with an acceptable burden of short-and long-term morbidity.

## Figures and Tables

**Table 1:** 

If there is marked global or regional atelectasis, consider:	
(i) Increasing PEEP to improve end-expiratory lung volume	
(ii) Increasing PIP to recruit atelectatic lung units	
(iii) Increasing inspiratory time to facilitate the recruiting effect of PIP	
(iv) Use of HFOV with sufficient distending pressure to recruit atelectatic lung units	
(v) Use of HFJV with sufficient PEEP to maintain FRC and conventional breath PIP to recruit atelectatic lung units	
(vi) Exogenous surfactant	
(vii) Lung lavage	

If there is obvious gas trapping, consider:	

(i) Decreasing PEEP (but may lose recruitment of areas prone to atelectasis)	
(ii) Decreasing inspiratory time and increasing expiratory time	
(iii) Use of HFJV with low PEEP, low frequency (240–360 bpm), and minimal CMV breaths	
(iv) Use of HFOV with relatively low *P*_AW_ and low frequency (5-6 Hz)	

If there is pulmonary hypertension, consider:	

(i) Correction of potentiating factors—hypoglycaemia, hypocalcaemia, hypomagnesaemia, polycythaemia, hypothermia, pain	
(ii) Bolstering systemic blood pressure to reduce right to left ductal shunt—volume expansion, pressor agents	
(iii) Improving right ventricular function—inotrope infusion	
(iv) Selective pulmonary vasodilators—inhaled nitric oxide	
